# Masculine attitudes of superiority deter men from accessing antiretroviral therapy in Dar es Salaam, Tanzania

**DOI:** 10.3402/gha.v6i0.21812

**Published:** 2013-10-22

**Authors:** Tumaini M. Nyamhanga, Eustace P.Y. Muhondwa, Rose Shayo

**Affiliations:** 1 Department of Development Studies, Muhimbili University of Health and Allied Sciences, Dar es Salaam, Tanzania; 2 Department of Behavioral Sciences, Muhimbili University of Health and Allied Sciences, Dar es Salaam, Tanzania; 3 Institute of Development Studies, University of Dar es Salaam, Dar es Salaam, Tanzania

**Keywords:** gender, masculinity, HIV, access, antiretroviral therapy (ART)

## Abstract

**Background:**

This article presents part of the findings from a larger study that sought to assess the role that gender relations play in influencing equity regarding access and adherence to antiretroviral therapy (ART). Review of the literature has indicated that, in Southern and Eastern Africa, fewer men than women have been accessing ART, and the former start using ART late, after HIV has already been allowed to advance. The main causes for this gender gap have not yet been fully explained.

**Objective:**

To explore how masculinity norms limit men's access to ART in Dar es Salaam.

**Design:**

This article is based on a qualitative study that involved the use of focus group discussions (FGDs). The study employed a stratified purposive sampling technique to recruit respondents. The study also employed a thematic analysis approach.

**Results:**

Overall, the study's findings revealed that men's hesitation to visit the care and treatment clinics signifies the superiority norm of masculinity that requires men to avoid displaying weakness. Since men are the heads of families and have higher social status, they reported feeling embarrassed at having to visit the care and treatment clinics. Specifically, male respondents indicated that going to a care and treatment clinic may raise suspicion about their status of living with HIV, which in turn may compromise their leadership position and cause family instability. Because of this tendency towards ‘hiding’, the few men who register at the public care and treatment clinics do so late, when HIV-related signs and symptoms are already far advanced.

**Conclusion:**

This study suggests that the superiority norm of masculinity affects men's access to ART. Societal expectations of a ‘real man’ to be fearless, resilient, and emotionally stable are in direct conflict with expectations of the treatment programme that one has to demonstrate health-promoting behaviour, such as promptness in attending the care and treatment clinic, agreeing to take HIV tests, and disclosing one's status of living with HIV to at least one's spouse or partner. Hence, there is a need for HIV control agencies to design community-based programmes that will stimulate dialogue on the deconstruction of masculinity notions.

Globally, antiretroviral therapy (ART) programmes have turned HIV from a virtual death sentence to a chronic and manageable disease (1, 2). At the close of 2008, more than 4 million people in low- and middle-income countries had gained access to antiretroviral drugs (ARVs), an increase from about 3 million in 2007 (3). Moreover, ART access in the countries of sub-Saharan Africa increased from 33% in 2007 to 44% in 2008 (4).

This is progress towards universal ART access in sub-Saharan Africa. However, it is considered marginal because more than half of the needy people living with HIV were not accessing ART by 2008. In view of these facts, it is important to understand which groups of people are particularly hard to reach or are not being reached effectively, and why this is the case.

The few studies of this topic that are available have indicated that, in Southern and Eastern Africa, fewer men than women have been accessing ART (5–8). In addition, other studies have shown that men start using ART late, when HIV is already advanced (9, 10). Similar observations have been made in Tanzania (11–14), but these studies fell short of explaining the cause of the gender gap. Simply asking who does and who does not receive particular services fails to capture how gender relations shape women's and men's access to and experience of treatment, care, and support services. These sources of information describe health inequities between men and women without examining the determinants of those inequities in their social context. It follows that the existing information in Tanzania does not shine much light on the specific determinants and consequences of the differences between men and women living with HIV with respect to accessing ART. This is because the central focus of these previous works in Tanzania was not on gender.

Previous studies (15–17) have documented barriers to accessing ART, including stigma and discrimination; non-disclosure; hunger, transportation, and opportunity costs; healthcare systems factors; non-disclosure; inaccurate knowledge and perceptions about HIV and ART; and certain religious beliefs and advice. However, few studies have attempted to capture the gender dimension of barriers to accessing ART.

## Theoretical framework

Accounting for gender is vital for understanding men's and women's differing health practices and illness experiences (18–20). The article is framed within Connell's (21) concept of hegemonic masculinity as applied by Courtney (22). Connell (21) argues that many cultures nurture a dominant (hegemonic) form of masculinity that demonstrates male power. Inherent in this concept of male power are the hegemonic qualities of a ‘real man’ – tough, fearless, and physically strong. In the process of illustrating their possession of the qualities of a real man, men tend to subordinate women and fellow men who have ‘feminine’ characteristics. It is the quest to maintain this powerful status that puts men's health at risk, as expounded by Courtney (22) in his relational theory of men's health. Courtney's theory derives its explanatory power from the premise that although several factors – such as socio-economic status and access to health services – are associated with health and longevity, they cannot fully explain gender differences in health and longevity (22). Health behaviours have been found to be important explanatory factors for the differences in health status between men and women. Health behaviours are in turn influenced by socio-cultural factors – most notably, by gender (23). As such, whereas women tend to exhibit more health-promoting behaviour, men have a tendency to engage in risky behaviours such as smoking, drinking alcohol, and careless driving (24, 25).

In spite of these gender-based differences in health behaviours, few studies have sought explanations for their causes. Social construction theorists (21, 22, 26–28) have argued that the gender-related social experiences of women and men provide a framework that guides their health beliefs and behaviours. Consequently, it is argued, men use the health behaviours that they exhibit as a means of constructing and demonstrating hegemonic masculinity – which places a high value on self-reliance, endurance, emotional strength, and fearlessness. Thus, ‘real men’ strive not to manifest ‘feminine qualities’ such as lack of endurance, emotional vulnerability, and promptness in seeking assistance (29). As a result, health behaviours are applied as part of men's strategies for negotiating a social landscape. It is therefore not surprising that a growing number of studies have established that men are less likely than women to access ART (8, 30–32). It is in this framework that we present and discuss findings from a broader study that sought, in part, to understand how gender-related factors influence men's access to ART.

## Methods

### Study setting and population

This article originates from a broader study conducted in Dar es Salaam from January to June 2009. The study sought to understand the role that gender relations played in influencing access and adherence to ART among men and women. The study population consisted of men and women living with HIV who were officially registered by nongovernmental organizations (NGOs) that support people living with HIV (PLWH).

The Dar es Salaam region was selected due to the fact that, as the largest city and economic centre of Tanzania, it has well-established care and treatment clinics and a range of active NGOs that provide support to PLWH. Respondents were recruited with the support of four NGOs – PASADA (Pastoral Activities and Services for People with AIDS, Dar es Salaam Archdiocese), SHDEPHA+ (Service, Health & Development for People Living Positively with HIV/AIDS), Tanzania Women Living with HIV/AIDS (TNW+), and the HIV/AIDS WAHANGA Group. The recruitment was purposely done through NGOs because they offered a non-threatening, non-judgemental atmosphere in which we could engage in discussions related to access and adherence to ART. A clinical setting, in contrast, was thought to be threatening in the sense that a power difference exists between service providers and clients. The providers expect ART users to exhibit 100% adherence to the treatment plan, and hence suboptimal adherence is considered a form of deviance. As a result, respondents may conceal their patterns of non-adherence to ART, thinking that somehow the providers may hear about this and become offended. Conversely, the NGO setting is non-judgemental in the sense that it is considered ‘home ground’ for people living with HIV.

### Sampling technique

The study used a stratified purposive sampling technique in order to illustrate the characteristics of men and women and to facilitate comparison between them with respect to access and adherence to ART (33). (Note that the larger study from which this article emanates sought to explore how gender influences access and adherence to ART – hence the reason for involving both women and men.) The strata of male and female focus group discussants who were recruited consisted of the following categories: unmarried, married, widows and widowers, and parents of children who were younger than 5 years of age. The leaders of select NGOs were approached and asked to assist in recruiting such respondents. After establishing the list of potential respondents, the leaders of the respective NGOs contacted them via telephone and invited them to participate in the study.

### Data collection

Data were collected using focus group discussions (FGDs) that consisted of groups of 6–7 respondents. The data collection process was led by two facilitators: a moderator and a note taker (recorder). A total of 12 FGDs were organized, including eight for women and four for men. There were 26 male and 52 female participants (Table 1).

**Table 1:** Characteristics of the focus group discussions conducted

	Number of sessions		Number of participants			
			
Category of FGD	Female	Male	Female	Male	Total
Unmarried, aged 15–24	1	–	6	–	6
Married, aged 25–45	3	2	19	13	32
Parent of under-5 child, aged 25–45	2	–	14	–	14
Widows and widowers	2	2	13	13	26
**Total**	**8**	**4**	**52**	**26**	**78**

The researchers encountered difficulty in recruiting male participants because of two factors. Firstly, participants were recruited through NGOs that support PLWH, and most of the members of such organizations are women. This does not mean that men living with HIV are few but rather – as the study found – that they shy away from assistance programmes. Secondly, even some of the men who were members of the support organizations could be hesitant to take part in group discussions because they needed to work to earn a living for themselves and their families. Again, this touches upon the question of the gender-based division of roles – all the more important in the urban setting of Dar es Salaam, where money is the key to meeting almost every basic need. Taking these factors into account, FGDs for men were scheduled on Saturdays in order to maximize their availability. The data were collected in Kiswahili, transcribed verbatim, and then translated into English.

### Facilitation of FGDs

The moderator was responsible for leading the FGDs, posing critical questions that were specified in the discussion guide, keeping the discussion on track, and making sure that all participants contributed to the discussion. For instance, a typical opening question was ‘How are women and men faring with respect to access and adherence to ART?’ In addition, participants were asked to give their views on factors contributing to the difference between men and women in access and adherence to ART. The probes included power differences in decision making, gender roles, gender-based ownership and expenditure of household resources, and gender dynamics in disclosure of HIV status.

### Ethical approval

Ethical approval was sought from the Institutional Review Board of Muhimbili University of Health and Allied Sciences. After the study received ethical clearance, permission to conduct the study was also sought and received from each of the three municipal authorities in the Dar es Salaam region – Kinondoni, Ilala, and Temeke. Each potential respondent was informed about the main objective of the study and about his or her right to decline participation outright, or to withdraw consent at any stage of the research, without undesirable consequences. If the potential respondent agreed to participate in the study, he or she was requested to sign a consent form.

Data were gathered without the researcher knowing the names of participants or being made aware of any unique identifiers attached to the data, although the names of the NGOs from which the respective participants had been recruited were not anonymized.

### Data analysis

A thematic analysis approach was used in analysing the study's findings, and the use of this approach implies that data were analysed through the examination and categorization of respondents’ opinions. The analysis was carried out in three stages (34, 35): firstly, the line-by-line coding of field notes and transcripts; secondly, the in-depth examination and interpretation of the resultant codes and their categorization into descriptive and analytical themes; and, thirdly, the development of an overarching theme (Table 2). The coding involved the development of concepts – that is, the data were parsed into discrete elements in order to expose underlying thoughts and meanings. The process generated 18 codes, which were further interpreted and categorized into eight descriptive codes. These latter codes were then further distilled into five abstract analytical themes, which are used as the headings in the “Results” section.

**Table 2:** Analytical framework: pathways through which masculinity interferes with men's access to antiretroviral therapy (ART)

Codes	Descriptive themes	Analytical themes	Overarching theme
Men do not want to show up. Shame Superiority Men are heads of family. Clinic is a women's space. Fear of degrading social status Embarrassment	Men consider themselves to have a higher social status. Men fear degrading the social status that they enjoy.	Social construction of masculinity	
Fear of being recognized by family members as living with HIV Men fear disclosing. Men fear stigma and discrimination. Fear of desertion	Men fear that going to the care and treatment clinic (CTC) would make their status of living with HIV known to their family members. Men fear that disclosure of their status of living with HIV may cause family instability – given their leadership position. Men are afraid of being deserted by their wives.	HIV perceived as a threat to men's masculinity	Masculinity interfering with men's access to ART
Fewer men at CTCs More women at CTCs Men steal their wife's antiretroviral drugs (ARVs). Preference of intermittent treatment of opportunistic infections (OIs) Buying ARVs from female clients Late clinic attendance Hesitant men die.	Out of shame, men suffer silently and attempt to cope by stealing their wives’ ARVs; seeking treatment of OIs; using their partner's doses, and/or buying ARVs from female clients. Men lag behind women in availing themselves of treatment at the CTCs. Men surrender and go to the clinic at an advanced stage of HIV.	Shame Men take advantage of their powerful decision-making and economic position to informally access ART. Unwillingness to admit problems	

## Results

All respondents – females and males – made several similar observations about the health-seeking behaviour of males. These centred on the concept of male superiority as inherent to masculinity in order to explain why men refrain from or hesitate to attend care and treatment clinics, even when their health is deteriorating. These observations are categorized into five analytical themes that emerged from all of the FGDs. They include *Social construction of masculinity*, *HIV/AIDS perceived as a threat to men's masculinity*, *Shame*, *Unwillingness to admit problems*, and *Men taking advantage of their powerful decision-making and economic positions to access ART informally*. An overall interpretation of these analytical themes gives rise to an overarching theme: masculinity interfering with men's access to ART. Specific findings are presented throughout this section. It is worth noting that the same FGD guide was used with both men and women. On this particular issue – how notions of hegemonic masculinity interfere with men's access to ART – both men and women had similar views. At the end of each quote, we have indicated who said it – a man or a woman.

### Social construction of masculinity

Respondents indicated that the influence of masculinity on men's access to ART stemmed from society's perception of what defines a man and how a man should behave. One participant said:

A man always strives to meet societal expectations of him. Being a man is being respectful, confident and fearless. (Married man, 42 years old)

In simple terms, this respondent was emphasizing the fact that men generally are expected to not be fearful and that it is through such beliefs that men construct their images of themselves as real men. Other participants added that a real man is an overseer of the family; he makes decisions and strives to ensure the welfare of his family.

### HIV/AIDS perceived as a threat to men's masculinity

The study findings indicated that men perceive HIV to be a threat to their masculinity due to the fact that men fear that disclosing their status of living with HIV may compromise their leadership position in the family and thereby cause or contribute to family instability. It was argued that, because a man is the head of and breadwinner for the family, his visiting a care and treatment clinic would amount to disclosing his status of living with HIV – which would, in turn, destabilize the remaining family members both psychologically and socially. Such sentiments were echoed in one of the FGDs:

Men fear having to explain openly about their positive serostatus. The family may become unstable. This is because after disclosing his positive serostatus people will immediately start stigmatizing him or the wife will desert him, among other undesirable outcomes. Thus, he sees that he should be firm so that his family does not stagger. (Widower, 38 years old)

As a result of the sentiment voiced in this extract, men often hesitate to register at the care and treatment clinics in an attempt to preserve their manhood – by ‘being firm’ and maintaining their emotional composure even when they are aware of their unhealthy status.

### Shame

Some respondents further indicated that, because men are the heads of families and have higher social status, they often feel embarrassed at having to visit a care and treatment clinic because they are afraid that it will negatively impact this status. One FGD participant aptly noted:

It is all about his status …. Men do not like to come out and have it be known that they are infected. They do not like it at all. One might have been tested and know his status, but he refuses to say, even though [he] is already infected. When it comes to going to the hospital, [he says] ‘Really should I go to the hospital and have it be seen that I am infected with HIV?’ He sees that he will be humiliated. (Married man, 27 years old)

As a result of this sentiment, it was reported that men are more likely to suffer in silence because of the effects of shame. This is because HIV – being considered a shameful disease – antagonizes men's expectations of being seen as dignified and worthy of respect. Several FGD participants asserted that some men do, in fact, avoid care and treatment clinics and instead seek treatment for opportunistic infections (OIs) from private health facilities, where they will not be seen by as many people:

A man may suspect he is infected with HIV but hesitate to go to the clinic for testing. He imagines that he will be humiliated. Consequently, if he has any opportunistic infection, he is ready to go to the private hospital for treatment and continue with his routine activities …. Really, men continue to hide! … Only a very small percentage of men are open. (Widower, 44 years old)

### Unwillingness to admit problems

Because of the shame-motivated tendency to ‘hide’, FGD participants observed that the few men who do register at the public care and treatment clinics tended to do so at a late stage, when their HIV-related symptoms were already advanced. Often, these are people have been very sick, to the extent of having to be admitted to a hospital, and after recovery they see no option but to surrender to the need to utilize the care and treatment clinics as a way to receive more regular treatment. That is, for men to agree to seek treatment, they must first go through a turbulent period of sickness. As one female participant put it:

If you see him at the hospital queuing together with you, then you should know that he had been seriously sick and even got admitted. Otherwise he would not go! (Mother of an under-5 child, 26 years old)

Another FGD participant similarly noted that men's decisions to visit the care and treatment clinics only late in the progression of their disease can be seen as a result of their ‘defiance’ – that is, their unwillingness to acknowledge promptly the fact that they need help and treatment. As a result, their physical appearance at the time they choose or are forced to visit care and treatment clinics causes them to appear to be sicker than their female counterparts:

If you go to the clinic and find men and women in a queue, the majority of women would look healthy. But a higher proportion of men look tired, debilitated by the disease. That is because of their defiance! (Mother of an under-5 child, 32 years old)

As a result, the FGD participants asserted that more men on ART die than do women on the same or a similar therapy. This assertion was supported by the clinic data (see Table 3) that were collected as part of the broader study.

**Table 3:** The trend of mortality among people living with HIV attending care and treatment clinics run by the Management and Development for Health (MDH) project, October 2004–December 2008

	Enrolment to care and treatment clinics		Number of deaths		Proportion of deaths against enrolment	
			
Year	Female	Male	Female	Male	Female	Male
2004 (October–December)	323	164	1	0	0.03	0
2005	5,383	2,238	413	305	7.7	13.6
2006	8,346	3,432	1,374	852	16.5	24.8
2007	10,111	4,379	1,847	1,221	18.3	27.9
2008	10,276	4,442	446	280	4.3	6.3
Total	34,439	14,655	45,36	2,658	13.2	18.1

Source: MDH Project, 2009

Table 3 shows that from the beginning of the care and treatment programme run by the MDH (Management and Development for Health) project in Dar es Salaam from October 2004 to 31 December 2008, a total of 4,536 women had died compared to only 2,658 men. This is true with respect to absolute numbers. However, examination of these numbers against total enrolment in the same period reveals that proportionally more men (18.1%) than women (13.2%) lost their lives. These deaths might be attributed to suboptimal adherence to and/or late starting of an ART regimen.

### Men taking advantage of their powerful decision-making and economic positions to access ART informally

It was further reported that some of those same men who tended to avoid visiting the care and treatment clinics instead opted to take their wives’ drugs or to buy drugs from women who had registered themselves at multiple care and treatment clinics for the express purpose of gaining access to extra ARVs. One FGD participant remarked:

Just as men are sluggish in going for VCT, they are also slow in accessing ARVs; thus some of them rely on using their wives’ doses or buy ARVs from people – particularly women – who have registered at more than one care and treatment clinic. (Unmarried young woman, 22 years old)

These practices – the taking or stealing of drugs prescribed to their wives and/or the buying of drugs from fellow patients – are dangerous to men's health, as they lead men to miss out on necessary medical attention.

## Discussion

### Construction of masculinity

Our findings suggest that the influence of masculinity on men's access to ART has stemmed from society's perception of what defines a man and how a man should behave. This is in agreement with Skovdal et al.'s (36) argument that socio-culturally rooted perceptions of ‘real men’ as strong, emotionally independent, tough, and fearless have a bearing on access and adherence to HIV treatment. She argued that these perceptions compel HIV-infected men to reassert their hegemonic masculinity by avoiding HIV-related services in order to illustrate that they are strong and resilient to illness. These implications of hegemonic notions of ‘a real man’ need to be addressed when mainstreaming gender in HIV care and treatment programmes. At the time of this study, Tanzania's National Multi-sectoral Strategic Framework on HIV/AIDS (NMSF) paid no attention to gender dimensions of care and treatment. That is, the NMSF addressed the gender question only as far as prevention of HIV transmission is concerned (37).

### HIV/AIDS perceived as a threat to manhood and inherent superiority

This study explored factors that explain why men in the Dar es Salaam region lag behind women in availing themselves of ART. Data indicate that feelings of superiority cause some men to feel ashamed at having to visit a care and treatment clinic in order to access the ARVs. It was argued that men are hesitant to utilize the public care and treatment clinics out of fear that doing so will degrade their superior social status. Similar findings have been noted in, for instance, Uganda and Zimbabwe (3, 14), where it was found that patriarchal norms that emphasize male supremacy affect men's access to ART. It was reported in these studies that men were more impacted by social stigma because they wanted to safeguard their position of supremacy and therefore could not be seen accessing services and thus being thought of as vulnerable. In addition, Skovdal et al. (38) report that their respondents thought that being ill undermines a man's sense of manhood and his role as head of household, and thus one would tend to avoid situations like clinic attendance that would expose his status of living with HIV.

Besides, by not disclosing their status of living with HIV, men put their spouses and partners at risk of infection or re-infection as they are less likely to practice safer sex. This kind of HIV transmission risk has also been reported in the United States (39).

### Men taking advantage of their powerful decision-making and economic positions to access ART informally

The findings of our study show that those men who tend to avoid visiting the care and treatment clinics opt to take their wives’ drugs or buy from fellow patients, particularly women who have registered themselves at more than one care and treatment clinic. This practice not only amounts to poor utilization of ARVs by men but also contributes to poor adherence among female users of ART. This is in agreement with findings reported by Skovdal et al. (38). Furthermore, buying ARVs from fellow patients suggests that there are flaws in the system of registering patients at the care and treatment clinics, such that it is possible for one to have double or triple registrations without being noticed. Similar findings have been reported in Uganda by Larsson et al. (40). The creation of an interconnected linkage between the care and treatment clinics may help to address the problem.

### Unwillingness to admit problems

Likewise, in this study FGD participants asserted that the men who are seen at public care and treatment clinics are typically those who had been very sick, to the extent of having to be admitted to a hospital. In other words, a number of men will visit a care and treatment clinic for the first time only when their HIV-related symptoms are already advanced. These findings substantiate the views of social construction theorists (21, 22, 26–28) that men use the health behaviours that they exhibit as a means of constructing and demonstrating hegemonic masculinity – which places a high value on self-reliance, endurance, emotional strength, and fearlessness. However, these masculinity-related social norms also influence men to engage in risky behaviours – including the decision to not avail themselves of ART (24, 25), or to do so late as reported by other scholars (12, 13).

As a consequence, this study found out through the statements of FGD participants and clinic data that proportionally more men on ART die than do women on the same or a similar therapy. Indeed, researchers whose project was running care and treatment clinics in Dar es Salaam at the time of the study published two papers indicating that men on ART had higher mortality than their female counterparts (41, 42). Similar findings have been reported elsewhere in sub-Saharan Africa (43–45).

### The intersection between services design and masculinity

This study has found that men do avoid going to the care and treatment clinics because doing so would amount to making one's HIV status known. The role that hegemonic notions of masculinity play in influencing the avoidance of care and treatment clinics by men is compounded by the design of the clinics. The municipal care and treatment clinics in Dar es Salaam are separated from the general outpatient department. This kind of arrangement reinforces stigma as men seen going there may be judged easily as living with HIV and as having probably started taking ART. Integrated provision of HIV care and treatment services might be an alternative solution. That is, HIV care and treatment services could be made part of ordinary services in the outpatient department.

### Study limitations

The findings of our study should be interpreted in the light of the strengths and weaknesses inherent to the use of group-based data collection methods. While FGDs are useful for exploring shared experiences, there are limitations on what individual group members can openly express in the presence of others (46). However, the researchers addressed this drawback by ensuring that the FGDs were conducted in a systematic manner. For instance, the moderator assured FGD participants of their confidentiality, and group members were asked to treat shared information as privileged and confidential. In addition, there were fewer men in the study, and the findings would have been stronger if the study sample had included men who had refused to begin or who had dropped out of ART. However, the men and women who did participate in this study enabled the generation of a rich body of data that has shed more light on how masculinity influences ART uptake among men. For a deeper understanding, future research could explore the first-hand views of men who refuse to begin or who have dropped out of ART.

## Conclusions

The findings of this study suggest that the superiority norm of masculinity affects men's access to ART. Societal expectations of a ‘real man’ to be fearless, resilient, and emotionally stable are in direct conflict with expectations of the treatment programme that one has to demonstrate health-promoting behaviour such as promptness in attending the care and treatment clinic, agreeing to take HIV tests, and disclosing one's status of living with HIV to at least one's spouse or partner. Men's failure to avail themselves of ART results in some of them adopting an unproductive coping strategy of using their spouse's or partner's doses of ARVs. This practice not only amounts to poor utilization of ARVs by men but also contributes to poor adherence among their spouses or partners. In view of these issues, it is recommended that there is a need for HIV control agencies to design community-based programmes that aim to stimulate a dialogue among men and, in particular, that show a means of deconstructing notions of masculinity that incorporate superiority in matters related to men's health. Moreover, the study has indicated that men's avoidance of care and treatment clinics is also attributable to the fact that the clinics are designed in a manner that reinforces stigma. Integrated provision of HIV care and treatment services might be an alternative solution.
